# Cost-benefit Analysis of IUI and IVF based on willingness to pay approach; case study: Iran

**DOI:** 10.1371/journal.pone.0231584

**Published:** 2020-07-14

**Authors:** Ali Darvishi, Reza Goudarzi, Viktoria Habib Zadeh, Mohsen Barouni

**Affiliations:** 1 Students’ Scientific Research Center (SSRC), Tehran University of Medical Sciences (TUMS), Tehran, Iran; 2 Department of Health Management and Economics, School of Public Health, Tehran University of Medical Sciences, Tehran, Iran; 3 Health Services Management Research Center, Institute for Futures Studies in Health, Kerman University of Medical Sciences, Kerman, Iran; 4 Afzalipour Clinical Center for Infertility, Afzalipour Hospital, Kerman University of Medical Sciences, Kerman, Iran; 5 Social Determinants of Health Research Center, Institute for Futures Studies in Health, Kerman University of Medical Sciences, Kerman, Iran; International Institute of Tropical Agriculture, NIGERIA

## Abstract

Assisted reproductive technologies (ARTs) are often considered luxury services by policy-makers and the general population, which are always susceptible of removal from public funding of health care. The analysis of the economic aspects of this scope seems essential due to the high prevalence of infertility in Iran and the high costs of infertility treatments. This study aimed to investigate the value put on IUI and IVF treatments by communities in Iran and the affordability of services based on community preferences. A cost-benefit analysis (CBA) was performed based on the WTP approach, and the contingent valuation method (CVM) was used to estimate WTP for IUI and IVF using a researcher-made survey in two cities of Kerman and Isfahan, Iran, in 2016–17. The sample size was 604, and the study sample frame to estimate WTP included two groups of couples who were/were not aware of their fertility statuses. The costs of one cycle of IUI and IVF were calculated according to the treatment protocols, tariffs of 2016–17, and medical information records of patients. The mean direct and indirect medical costs of one cycle of IUI and IVF were equivalent to 19561140 and 60897610 IRR, respectively. Also, the mean WTP for IUI and IVF treatments were obtained of 15941061 and 28870833 IRR, respectively. The demand for IUI and IVF treatments was elastic and the community was sensitive to price changes of these treatment methods. IUI and IVF treatments brought no positive net benefits, and economic variables had the highest impact on the WTP and community preferences, indicating the significant role of financial constraints in the community's valuation for advanced infertility treatments in Iran.

## Introduction

Recently, infertility among couples has become one of the major problems in many countries [1]. Worldwide prevalence of infertility varies between 8 and 12% among reproductive-aged couples [2], and it has been estimated that about 70 million couples in the world are suffering from infertility [3, 4]. The last statistical estimate of the national program shows a prevalence of 20% for infertility in Iran [5].

Intrauterine Insemination (IUI) and In Vitro Fertilization (IVF) are types of Assisted Reproductive Technologies (ART) used to empower infertile couples to experience a successful pregnancy [6, 7]. IUI is one of the widely applied infertility treatment methods for infertility with unknown factors and with male problems (some sperm disorders). Also, it can be used for the infertility problems caused by the condition of the cervical mucus and sexual disability [8]. IVF is the most popular and advanced ART method, which was performed for the first time in 1977 and led to the birth of Louise Brown in 1978 [9]. An IVF cycle typically involves ovarian stimulation, followed by the retrieval of multiple mature oocytes fertilized in the laboratory to create embryos. The likelihood of the success of ART methods depends on different variables. For example, the chance of having an embryo in one IVF cycle depends on the patient's age, the cause of infertility, and the history of infertility treatments (but generally estimated at 20% per cycle) [10].

Despite the successes and advances of ART techniques (such as IVF and IUI) in the treatment of infertility, only 22% of infertile couples have received these services [11]. Although there are complex reasons for the inconsistency between the prevalence of infertility and the extent of treatment-seeking, the cost of ARTs is a severe barrier [12–14].

Organizing, presenting, and providing appropriate financial health services for the whole society are nowadays one of the principal goals of governments around the world [15]. In many developing countries, inadequate funding of the health sector is a major problem [16]. ARTs are typically assumed as expensive services by policy-makers and the general population, which are always prone to be removed from public funding of health care. In the short term, removal of the high costs of infertility treatments and ART services might be beneficial to the government. However, in the long run, a great wish of couples to have children will come true by the birth of each individual using these services. In other words, the newborn child will become an economically active adult in the community and generate income, including tax revenues for government and society [17]. Therefore, health economists have often paid much attention to economic outcomes of health, financial costs, and financial medical interventions—analyzed in terms of different economic evaluations, such as cost-benefit analysis (CBA), cost-effectiveness analysis (CEA), and cost-utility analysis (CUA).

Economic evaluation measures, such as quality-adjusted life years (QALY), are used to calculate the value of the health interventions’ outcomes that improve the health status of individuals. Given the difference of infertility interventions in the type of outcome (here, generation of a new life), it would not be possible to use common metrics to calculate the value of their outcome. An alternative approach to solving this problem is to use a currency unit for the outcome, which is called monetary units in the form of cost-benefit analysis (CBA). CBA is particularly beneficial for valuing non-market goods and services that exhibit both health and non-health outcomes [18]. Since the production of a new life is an excellent example of non-market goods, the analysis of the outcome values of infertility treatments using monetary units and cost-benefit analysis seems to be a practical solution. A common method for measuring the value of non-market goods or outcomes is expressed preferences and the willingness to pay, which reflects the total utility gained from all health and non-health outcomes [19, 20].

In Iran, it is vital to pay a great deal of attention to the worthy population of infertile couples due to the high prevalence of infertility, decreasing population growth rate, and population tendency toward aging on the one hand and the focus of government policies on population growth on the other hand. In this regard, one of the levers to enhance population growth in Iran is the spread of infertility treatments to increase the birth rate. However, before implementing such a plan, it is highly required to obtain scientific evidence of the community's preferences for these advanced treatments and specify the exact costs of these treatments from the first visit to the end of treatment. Thus, this economic-evaluation study is performed for the first time in Iran, aiming to provide scientific evidences using CBA to promote the necessary awareness of the potential market status of IUI and IVF and assess the value put on these treatments by the community in Iran and affordability of these services based on the community preferences.

## Materials and methods

### Study design and sampling

The present study is a cross-sectional descriptive-analytical investigation aiming to carry out a full economic evaluation in the health care system, which has been done using available field data in two cities of Kerman and Isfahan, Iran, in 2016–17. These two cities were selected since, according to the latest official statistics on the prevalence of infertility in Iran, Kerman province has the highest prevalence of infertility, and Isfahan province is among the five regions with the highest prevalence of infertility. Moreover, there are significant differences between the two cities in the culture, society, and economic behavior of citizens towards the demand for goods and services.

The study sample to estimate benefits included two groups. The first group was comprised of couples who were not aware of their fertility status and had no child until the study (ex-ante perspective). Data on this sample were collected from individuals of Kerman and Isfahan pre-marriage counseling centers and couples who referred for pre-marriage education. The second group included couples who were aware of their fertility status and had no child either (ex-post perspective). For greater and better access to all sectors of society in terms of socio-economy, half of the samples were collected from the private specialized centers and the other half from the educational public hospitals in both cities. In this regard and accordance with the approach adopted in this study for WTP, the table proposed by Mitchell and Carson was used for sample size estimation [21]. This table shows the minimum sample required for different levels of confidence and acceptable error in contingent valuation method (CVM) for measuring WTP. In this study, this table was used to estimate the sample size with a relative error of 1.5 and a confidence coefficient of 0.05, which resulted in a total sample size of 604 individuals. A total of 638 interviews were conducted to compensate for the loss to follow-up probability, where 620 couples had the inclusion criteria and entered the study. The sampling method was systematic and interviews were performed with 4 interval sizes.

### Estimation of costs

Total records on IUI and IVF in private and public centers were investigated and the number of used services in IUI and IVF treatment processes was recorded in each case separately, due to the limited number of medical records of patients in infertility clinics in 2015–16, to estimate the costs of IUI and IVF and reach higher accuracy of costing. The total numbers of IUI and IVF records were 197 and 294, respectively. The cost of one IVF cycle from the first visit to the last stage was calculated using experts’ medical information, treatment protocols, and medical records of patients in infertility clinics based on tariffs in private and public sectors from the relative value book of 2016–17.

To accurately calculate costs per capita of one IUI and IVF cycle, at first, information of treatment costs, medicines, and different medical services (such as laboratory and diagnosis services, medications, and specialized medical services) from the first visit to the last stage were evaluated based on protocols and views of gynecologists, and then information was recorded in a checklist. Medical records of the infertile patients in Afzalipour public clinical center and the private specialized infertility clinic of Najmiyeh, Kerman, were used to calculate mean consumption of medications and medical services in both IUI and IVF cycles. In the steps of the treatment protocol with patients’ variable use of drugs and services (such as the number of ultrasonography and injections), medical records of patients were examined and the average usage was taken into account for the cost evaluation (recorded in a checklist). After producing the checklists and performing accurate price evaluation for medicines and medical services using private and public medical centers tariffs, the costs of IUI and IVF were separately calculated.

The process of costing was as follows. After completing the special checklist, the treatment protocols were firstly applied, and then all 197 IUI and 294 IVF medical records in 2015–16 in two infertility treatment centers were evaluated to obtain the average consumption of medications and services that were variable among different patients (such as the number of ultrasonography and injections). Mean direct medical costs of one cycle of IUI and IVF were calculated after a review of all the measures, diagnostic and laboratory services, medications, specialized services from the first visit to the last stage of treatment based on treatment protocols, the relative value book, and also the cost inquiry from pharmacies and public/private infertility centers.

### Estimation of benefits (WTP)

WTP approach was used for the calculation of benefits, which is the main principal and the most valuable method for computing monetary benefits of health interventions in CBAs [20, 22]. A researcher-made questionnaire was designed to calculate people WTP for IUI and IVF cycles. The contingent valuation method (CVM) and the bidding game technique were used to measure the WTP. CVM is a survey method for valuing goods and services that is also applied to health interventions, in which participants are asked to specify their maximum WTP for a service using hypothetical scenarios [23, 24]. For collecting data on WTP, two interviewers were conducted in each city. As the researchers were very sensitive about the interview methods and their quality, the items were taught to interviewers in two workshop sessions. For this purpose, different scenarios were described under certain assumptions that asked for the maximum WTP of individuals. Also, one question was defined for each scenario about the willingness to accept (WTA) if WTP of the interviewed person was zero, as follows: “now that your WTP for this service and its success chance is zero, how much money do you wish to receive to accept the financial risk of disease with the conditions of this scenario?”. It is worth noting that based on the theory, the amounts of WTA must be subtracted from WTP [25].

#### The protocol of WTP estimation

A researcher-designed WTP protocol was used to estimate benefits and measure WTP of people in this study. The protocol was designed in three parts as follows. The first part included the demographic information on infertility and prevalence of infertility, as well as other medical and economic information on infertility and the purpose of the study—which were explained to the interviewees by the interviewer. It should be noted that, before the interview process begins, informed consent was obtained from the respondents in written form at this stage.

The second part was related to questions about WTP of IUI and IVF, in which the overall information of these treatment methods and mean costs were explained to the interviewees. Also, 4 scenarios of WTP with different chances of treatment success (10, 25, 50 and 99%) were considered. WTP was estimated by the open-ended bidding game in each scenario. For example, one of the scenarios is as follows:

Assume that you are infertile and can be treated with IUI/IVF treatment cycles, while the success rate of treatment is 25%, so how much are you willing to pay for it? Are you willing to pay X IRR for it?

If he/she accepted the prices of the initial point, higher prices were suggested; otherwise, the interviewer suggested lower prices, and these questions continued for 4 price points. Finally, an open question was asked about the ultimate willingness to pay for this service. Also, between the 4 proposed price points, some open price suggestions were asked to increase the accuracy of the WTP results. Also, for each scenario, one question was set on the willingness to accept (WTA) that was asked if WTP was zero.

Moreover, the third part of the protocol was about the demographic and socio-economic information of interviewees, which contained 18 questions.

Also, a pilot study was conducted on 30 couples in Kerman to detect and correct the probable shortcomings and error of the protocol.

The economic parts and procedures were validated by economic evaluation experts. Also, the clinical aspects of this method were validated by physicians and experts in the field of infertility from Kerman University of Medical Sciences. The Protocol was approved by ethics committee of Tehran University of Medical Sciences (TUMS) by the code of IR.TUMS.NIHR.REC.1395.8.

#### Extraction of demand functions

In this study, the demand functions of IUI and IVF were extracted from the corresponding individuals’ WTP. According to the theories of economics, a demand function reflects the maximum WTP of people to receive a certain amount of goods and services. Also, the relationship between quantity and price of a product, under normal conditions, obeys the law of downward sloping demand, i.e., the demand for the product or service decreases by increasing the prices [26]. The important assumption in the extraction of demand function is that when the person has accepted the high-price recommendations, he has indeed accepted the lower prices as well. In this regard, the demands for the IUI and IVF treatment methods were calculated at different price levels to extract their linear demand function, as given by Eq (1).

$$ln\;Q_{i}=\alpha ‐\beta \;ln\;P_{i}+\varepsilon$$ (1)

Where Q represents the amount of demand (number of people accepting the proposed price points), *P* denotes the proposed and accepted prices (WTP), *β* stands for the slope of the demand function, *α* is the intercept of the demand function, and ε is the statistical error. Moreover, all data on prices and amounts were initially changed to logarithmic form (here, *β* is indicative of demand price elasticity) to provide a better and more accurate estimation of the demand function and calculation of price elasticity of demand.

### Economic analysis of WTP/CBA

Ordinary least square (OLS) and weighted least squares (WLS) regression (techniques of econometrics) were used to estimate the demand function of IUI and IVF. Also, the steps of these two methods, as well as *Breusch Pagan* test for heteroskedasticity of variances, were performed by Stata software version 14.

Also, regarding the obtained WTP and costs, the CBA of one cycle of IUI and IVF was calculated. These calculations were used to perform a correct analysis of budget allocation of scarce resources. For a positive net benefit in a project, the distribution of resources and investment in the project were prioritized. The process of calculating CBA is usually performed by two indices, including benefit-cost ratio (BCR) and net present value (NPV) [25]. In this study, both indices were used (Eqs (2) and (3)).

$$NPV=\sum_{0}^{n}\left(\frac{Benefits_{\left(t\right)}-Costs_{\left(t\right)}}{(1+{r)}^{t}}\right)$$ (2)

$$BCR=\frac{PV(benefits)}{PV(costs)}$$ (3)

In Eqs (2) and (3), PV represents the present value and t denotes years. Also, r is indicative of the discount rate used to change the upcoming year’s costs and benefits to the current rate and omit the value of currency changes over time. The NPV equation was designed assuming that some costs and benefits were related to future years, where all prices were converted to the present currency value.

## Results

### Descriptive statistics

As already stated, 620 individuals were interviewed using the designed WTP questionnaire. The sample characteristics are listed in Table 1. As can be seen, the respondents were 29 years old on average, and 56% of interviewees were female. Also, the average family income of study respondents was about 22,780,000 IRR. About 50% of participants received university educations, 92% of respondents had basic health insurance, and the employment rate was about 59%.

**Table 1:** Sample characteristics.

*No*. *of respondents*	620	
*Mean age in years(Range)*	29.7(18–54)	
Average Household income (IRR)*	22780000	
	n	%
***Gender****(Female)*	348	56.2
***Education Level***		
■ Elementary and Secondary education	97	15.6
■ High school diploma	213	34.4
■ Associate Degree and Bachelor's Degree	246	39.7
■ Master's degree or higher	64	10.3
***Insurance status***		
■ Basic health insurance	573	92.5
■ Supplementary health insurance	210	33.8
■ History of infertility treatments	199	32.1
***Employment status***		
■ Employed	366	59.1
■ Student	23	3.7
■ Housekeeper	214	34.5
■ Unemployed	6	0.9
■ Other (soldier, etc.)	11	1.8

*The currency rate of 1 PPP $ in the study year was equivalent to 8256.8.

### Analytic results

In this section, the analytic results are explained individually, including cost per cycle of treatment methods, WTP, analysis of CBA and the demand functions for IUI and IVF.

Costs. Based on the cost items presented in Table 2, the cost of one cycle of IUI and IVF from the first visit to the end stage was calculated. Mean direct medical costs of one cycle of IUI and IVF were 19561140 and 60897610 IRR, respectively.

**Table 2:** IUI and IVF cost items and services.

Medical classification and subjects	Usage	
***Diagnostic and laboratory services***		
■ Visit	IVF	IUI
■ Intravascular ultrasound	IVF	IUI
■ Spermogram	IVF	IUI
■ blood test	IVF	IUI
■ Pap smear test	IVF	
■ Antigen test	IVF	
■ Injection	IVF	IUI
***Specialized medical services***		
■ Ovarian puncture	IVF	
■ Sperm washing	IVF	IUI
■ IUI		IUI
■ Transfer	IVF	
***Medicine and Medications***		
■ Clomiphene citrate	IVF	IUI
■ HMG	IVF	IUI
■ HCG 5000	IVF	IUI
■ Buserelin (CinnaFact)	IVF	
■ Projestronum	IVF	
■ Syringe	IVF	IUI

#### Benefits (WTP)

The mean WTP values for IUI in the scenarios 1 to 4 were 6156182, 10360752, 15746235, and 31501075 IRR, respectively. Mean WTP for IUI treatment was obtained from mean WTP of all respondents for the four scenarios as 15941061 IRR per cycle of IUI. Similarly, the mean WTP values for IVF in the four scenarios were 7666129, 17966666, 31810215, and 58040322 IRR, respectively and Mean WTP for IVF treatment was obtained from mean WTP of all respondents for the four scenarios as 28870833 IRR per cycle of IVF. Tables 3 and 4 list the amounts of WTP per one cycle of IUI and IVF, respectively.

**Table 3:** The costs and benefits of IUI per cycle in 4 scenarios.

Subject	Scenario 1 (10%chance of success)	Scenario 2 (25% chance of success)	Scenario 3 (50% chance of success)	Scenario 4 (99% chance of success)	Mean
WTP (IRR)*	6,156,182	10,360,752	15,746,235	31,501,075	15,941,061
Cost per cycle of IUI (IRR)*	19,561,140	19,561,140	19,561,140	19,561,140	19,561,140
NPV(IRR)*	-13,404,958	-9,200,388	-3,814,905	11,939,935	-3,620,079
BCR	0.31	0.52	0.80	1.61	0.81

* Data were presented on average and the currency rate of 1 PPP $ in the study year was equivalent to 8256.8.

**Table 4:** The costs and benefits of IVF per cycle in 4 scenarios.

Subject	Scenario 1 (10%chance of success)	Scenario 2 (25% chance of success)	Scenario 3 (50% chance of success)	Scenario 4 (99% chance of success)	Mean
WTP (IRR)*	7,666,129	17,966,666	31,810,210	58,040,320	28,870,833
Cost per cycle of IVF (IRR)*	60,897,610	60,897,610	60,897,610	60,897,610	60,897,610
NPV(IRR)*	-53,231,481	-42,930,944	-29,087,400	-2,857,290	-32,026,777
BCR	0.12	0.29	0.52	0.95	0.47

*Data were presented on avergae and the currency rate of 1 PPP $ in the study year was equivalent to 8256.8.

### Economic analysis of WTP/CBA

CBA of IUI and IVF. NPV and BCR of IUI and IVF treatments represented negative net benefits. In other words, from the community perspective, the value of these treatment methods was less than their costs, indicated by the numerical calculations of the two following equations. Also, these calculations for the four scenarios were done separately (Tables 3 and 4). The interesting point in CBA of IVF treatment was attributed to the results of NPV and BCR representing negative net benefits, even in scenario 4 with the assumption of treatment success chance of 99%.

#### The demand function for IUI and IVF

The results of the *breusch pagan* test for the IUI demand function showed heteroscedasticity of variances in the OLS regression (P-value = 0.0002 | χ^2^ = 13.95). tThe results of this test for the IVF demand function also was similar (P-value = 0.0429 | χ^2^ = 4.10). The WLS method was used according to the economic theories to resolve the heteroscedasticity of variances, as shown in Tables 5 and 6.

**Table 5:** Demand function for IUI treatment using WLS model.

The Explanatory variables	*β*	*SE*	*P-value*
**Intercept**	26.17	1.39	0.000
**Proposed Price**	-1.48	0.09	0.000
**Goodness of fit indexes**			
R^2^*	0.86		-
F*	234.86		0.000

* Data Evaluated by WLS Regression and Independent t-test, and p<0.05 shows the significance.

**Table 6:** Demand function for IVF treatment using WLS model.

The Explanatory variables	*β*	*SE*	*P-value*
**Intercept**	24.64	2.38	0.000
**Proposed Price**	-1.32	0.16	0.000
**Goodness of fit indexes**			
R^2^*	0.71		-
F*	68.19		0.000

* Data Evaluated by WLS Regression and Independent t-test, and p<0.05 shows the significance.

According to the economic theories, as the logarithmic form of data was used to estimate demand function, *β* of the proposed price and the slope of demand function reflect the demand elasticity for both treatment methods. Since *β* values were -1.48 and -1.32 for IUI and IVF functions, respectively, it shows that demand for these treatments was elastic in this study. In other words, as the price of one cycle of IUI and IVF treatments increased by one percent, the amounts of their demands were reduced by 1.48% and 1.32%, respectively.

**Fig 1:**
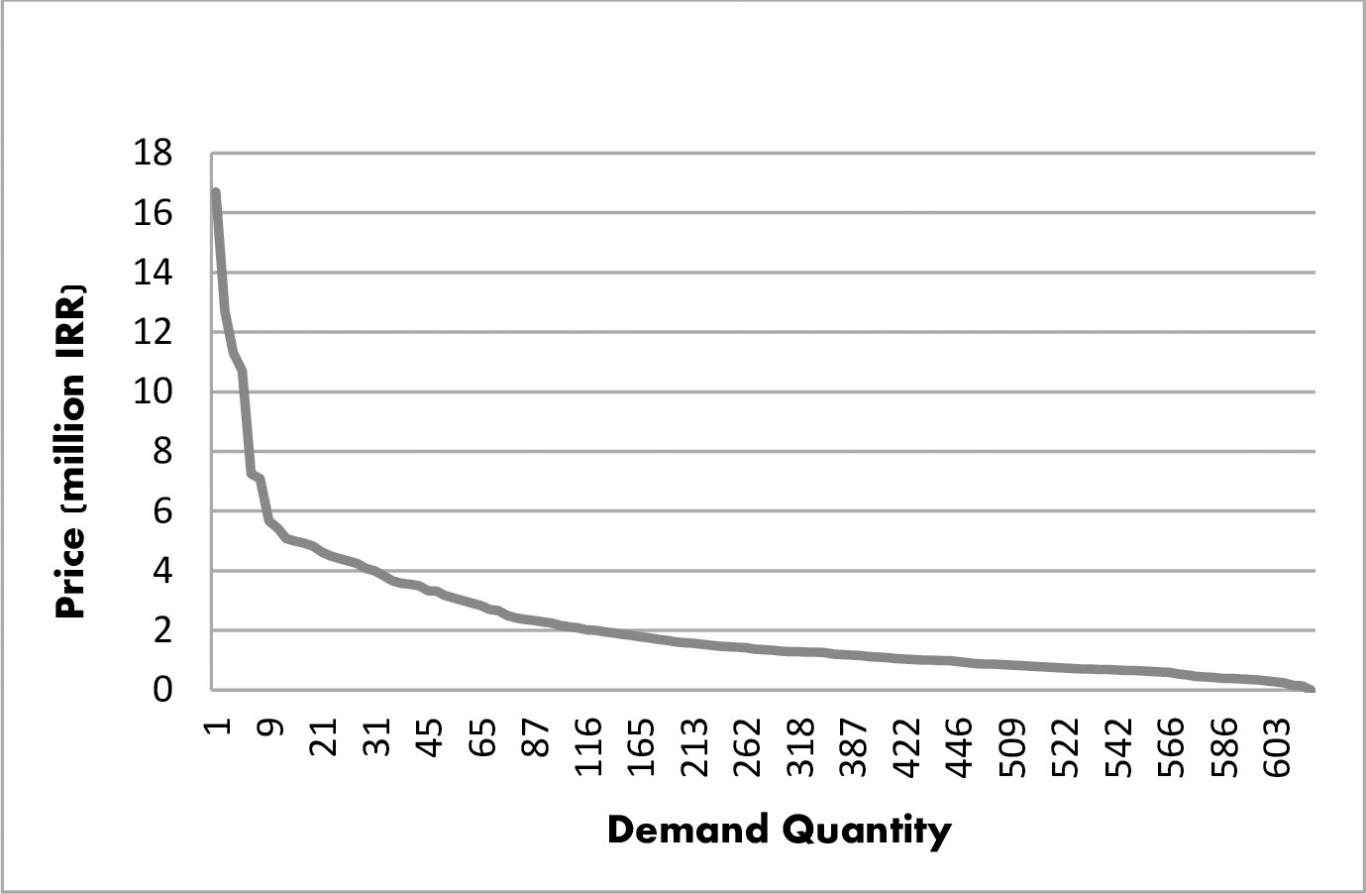
IUI demand curve.

**Fig 2:**
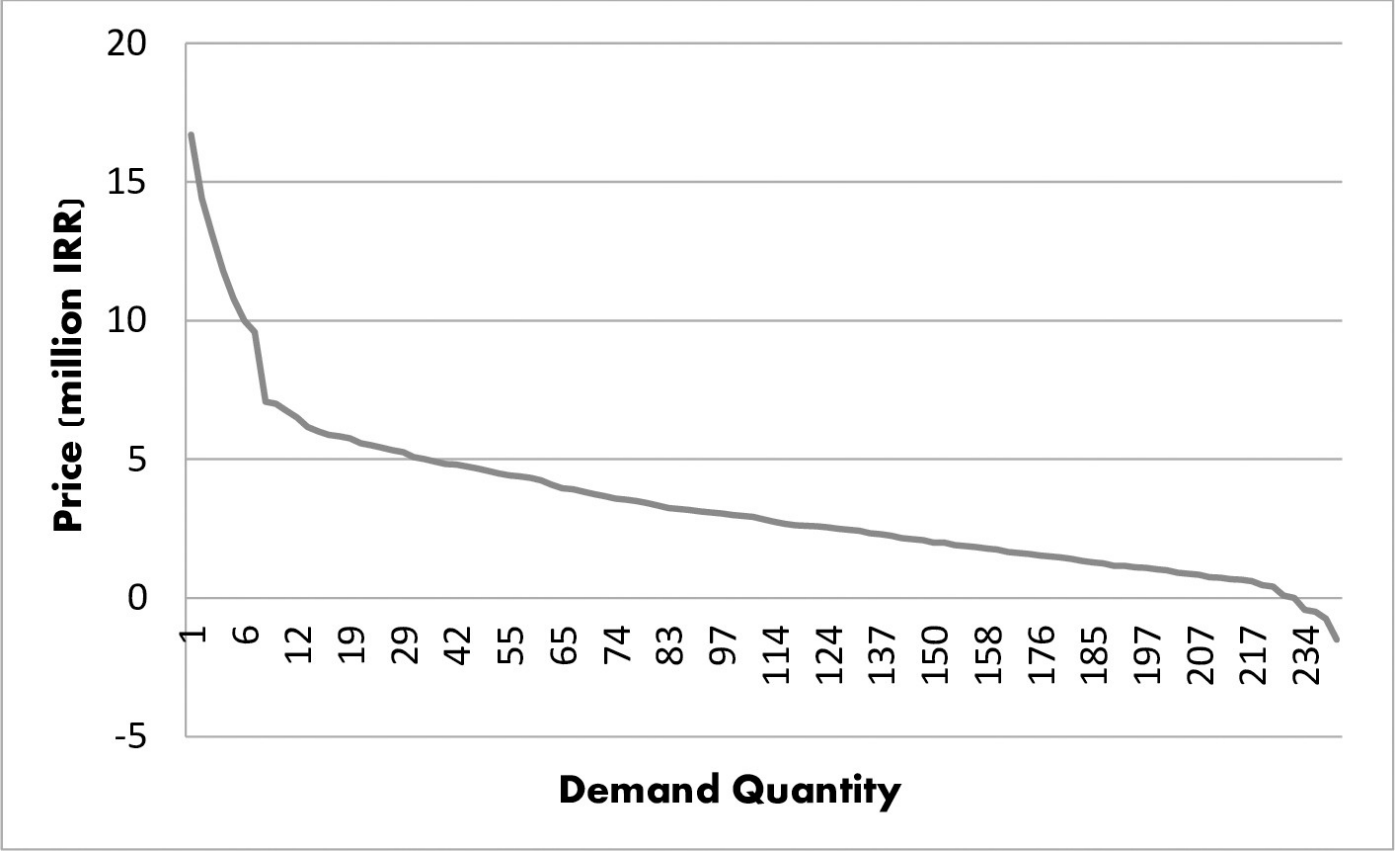
IVF demand curve.

Concerning the cross-sectional nature of the data, the amount of R^2^ showed good fitness of the selected model for both functions (IUI: R^2^ = 0.86, IVF: R^2^ = 0.71). Also, F statistics showed the significance of the total regression. The Demand function Curve for one cycle of IUI and IVF treatment was extracted as shown in Figs 1 and 2.

## Discussion

Analytic results showed that costs of one cycle of IUI and IVF treatments were 19,561,140 and 60897610 IRR with WTP of 15,941,061 and 29535410 IRR, respectively, which indicated that WTP was significantly less than the real costs of one cycle of IUI and IVF and showed lack of positive net benefits as it came from NPV and BCR results. The results of the study conducted by Spiegel et al. (2013) are contrary to the results of the present research. In their study, mean WTP of the patients for one cycle of IVF was $5482, and the WTP of the general public was $4398. Both WTP amounts were higher than the mean cost of one cycle of IVF in Israel ($3257) [27]. Results of the study performed by Settumba et al. (2018) in Australia that was based on ex-post perspective showed that the mean WTP for one IVF cycle ranged from $6135 to $13,561, whereas mean WTP for one year of IVF treatment varied from $17,080 to $31,006 [28].

The results of WTP of IUI and IVF based on each scenario indicated that WTP of couples was sensitive to chances of treatment success. In other words, a higher success chance of treatment increased the WTP of the community. Also, the study conducted by Neumann and Johanessonn (1994) in the United States on WTP for IVF validates the results of the present study [29].

Despite low WTP for IUI and IVF and lack of positive net benefits in the present study, as well as regarding to the demographic structure of Iran, there is still a relative pessimism among the general public about these advanced techniques. Nevertheless, these therapies have been used for many years in Iran. Also, lower levels of community WTP might be attributed to the time-consuming treatments and their difficult process and relatively indifinite success rate. Although the advanced infertility treatments have existed for several years in Iran, the general population has not had sufficient knowledge and correct views about them, which entails more awareness in this regard. Secondly, the low level of WTP can be due to low levels of population income on the one hand and a relatively high cost of the treatments on the other hand. Spiegel et al. (2013) concluded that one unit increase in monthly income has a positive effect in the amount of WTP for IVF [27]. Also results of Neumann and Johanessonn (1994) study showed that expected income had a significant positive effect on mean WTP for IVF in general public [29].

Moreover, based on the results of the present study, the elasticity of demand for IUI and IVF treatments was high, i.e., the community was sensitive to price changes of IUI and IVF. These results also indicated that the cost of infertility treatments was the most important factor in community demand. Hence, financial support seems to be the most effective factor in stimulating the community's demand for these therapies. Also, in order of priority, other parameters include promoting public awareness about these advanced therapies and changing traditional attitudes.

Furthermore, the viewpoint on economic issues of infertility, either long-term or short-term, is a crucial element. Analysis and conclusions based on each of these perspectives will change the policies and their implementations. For the short-term economy viewpoints, financial support, subsidy, and investment in costly ARTs for the treatment of infertility had no positive net benefits. However, from the long-term economic viewpoint, children born through this methods would be an economically active person in future, especially in Iran's current situation which is threatened by the risk of reducing workforce and population aging in the future. Accordingly, if the long-term vision and other social goals associated with having a child of infertile couples are considered, financial covering these treatments by the government can be very beneficial for the country. Discussion about financing these treatments and their stability are important issues as well. For hasty and sensational policies, perhaps the results contrary to our goals will be witnessed in the future. In the present study, the mean amount of WTP for IUI and IVF was estimated, and accurate costing was conducted, so the evidence of the study can be very useful and practical in this regard.

The strengths of this study, compared with similar studies in the world, can be the accurate costing based on the treatment protocols from the first visit to the final stage of IUI and IVF in Iran. The present study is one of the first economic studies in the field of infertility treatments in Iran. Also, it is one of the first economic evaluation studies that measured WTA along with WTP in this field. This study was carried out using the most complete CVM and attempted to eliminate the structural bias of this method as far as possible. The use of different initial points and open questions were among the points suggested for a more accurate estimation of the WTP. The sample frame of the study for the selection of people who were not aware of their fertility statue was a limitation since couples that refer for the Pre-marriage counseling centers cannot fully explain the characteristics of the general public and non-patients.

## Conclusions

Study results showed that the real costs of two advanced infertility services was more than their benefits from the social perspective of Iranians, and the most important factors in this regard were economic variables include low average expected income and relatively high costs of these ART services. Accordingly, in line with population growth policies in Iran, government support of infertile couples seems to have a significant impact on encouraging couples to use infertility services. Future studies using alternative scientific approaches as well as other ART services and broader geography consideration could provide more comprehensive and accurate evidence.

## Supporting information

S1 Fig Original questionnaire (Persian). (DOCX)

S2 Fig Translated questionnaire (English). (DOCX)

## References

[1] Mbizvo MT, Chou D, Shaw D. Today's evidence, tomorrow's agenda: implementation of strategies to improve global reproductive health. International Journal of Gynecology & Obstetrics 2013;121:S3–S8. 23490425 10.1016/j.ijgo.2013.02.007

[2] Sonaliya KN. Infertility: Ongoing Global challenge of new millennium. Indian Journal of Community Health 2016;28(2):113–5.

[3] Inhorn MC, Patrizio P. Infertility around the globe: new thinking on gender, reproductive technologies and global movements in the 21st century. Human reproduction update. 2015;21(4):411–26. 10.1093/humupd/dmv016 25801630

[4] Mascarenhas MN, Flaxman SR, Boerma T, Vanderpoel S, Stevens GA. National, regional, and global trends in infertility prevalence since 1990: a systematic analysis of 277 health surveys. PLoS medicine 2012;9(12):e1001356 10.1371/journal.pmed.1001356 23271957 PMC3525527

[5] Akhondi MM, Kamali K, Ranjbar F, Shirzad M, Shafeghati S, Ardakani ZB, et al Prevalence of primary infertility in Iran in 2010. Iranian journal of public health 2013;42(12): 1398–1404. 26060641 PMC4441936

[6] Edwards RG, Sharpe DJ. Social values and research in human embryology. Nature 1971;231(5298): 87–91. 10.1038/231087a0 4930102

[7] Berek JS, Hillard PJA. Berek & Novak’s Gynecology. 14th Edn, 2007 Lippincott Williams & Wilkins, US.

[8] Campana A, Sakkas D, Stalberg A, Bianchi PG, Comte I, Pache T, et al Intrauterine insemination: evaluation of the results according to the woman's age, sperm quality, total sperm count per insemination and life table analysis. Human Reproduction 1996;11(4):732–6. 10.1093/oxfordjournals.humrep.a019244 8671318

[9] Brezina PR, Zhao Y. The ethical, legal, and social issues impacted by modern assisted reproductive technologies. *Obstetrics and gynecology international* 2012;1–7. 10.1155/2012/686253 PMC3261493 22272208

[10] Fitzgerald O, Paul RC, Harris K, Chambers GM. Assisted reproductive technology in Australia and New Zealand 2018 National Perinatal Epidemiology and Statistics Unit, the University of New South Wales Sydney, Sydney.

[11] Boivin J, Bunting L, Collins JA, Nygren KG. International estimates of infertility prevalence and treatment-seeking: potential need and demand for infertility medical care. Human reproduction 2007;22(6):1506–12. 10.1093/humrep/dem046 17376819

[12] Chambers GM, Sullivan EA, Chapman MG, Ishihara O, Zegers-Hochschild F, Nygren KG, et al The impact of consumer affordability on access to assisted reproductive technologies and embryo transfer practices: an international analysis. Fertility and sterility 2014;101(1):191–8. 10.1016/j.fertnstert.2013.09.005 24156958

[13] Jones HW Jr, Cooke I, Kempers R, Brinsden P, Saunders D. International Federation of Fertility Societies Surveillance 2010: preface. Fertility and sterility 2011;95(2):491 10.1016/j.fertnstert.2010.08.011 20813358

[14] Marcus D, Marcus A, Johnson A, Marcus S. Infertility treatment: when is it time to give up? An internet-based survey. Human Fertility 2011;14(1):29–34. 10.3109/14647273.2010.541971 21329471

[15] Devlin RA, Sarma S, Zhang Q. The role of supplemental coverage in a universal health insurance system: Some Canadian evidence. Health policy 2011;100(1):81–90. 10.1016/j.healthpol.2010.08.011 20810187

[16] Gustafsson-Wright E, Asfaw A, van der Gaag J. Willingness to pay for health insurance: An analysis of the potential market for new low-cost health insurance products in Namibia. Social science & medicine 2009;69(9):1351–9. 19765877 10.1016/j.socscimed.2009.08.011

[17] Moolenaar L, Connolly M, Huisman B, Postma M, Hompes P, van der Veen F, et al Costs and benefits of individuals conceived after IVF: a net tax evaluation in The Netherlands. Reproductive biomedicine online 2014;28(2):239–45. 10.1016/j.rbmo.2013.10.002 24365025

[18] Fawsitt CG, Bourke J, Murphy A, McElroy B, Lutomski JE, Murphy R, et al A cost-benefit analysis of two alternative models of maternity care in Ireland. Applied health economics and health policy 2017;15(6):785–94. 10.1007/s40258-017-0344-8 28828573 PMC5701951

[19] Ryan M. Using conjoint analysis to take account of patient preferences and go beyond health outcomes: an application to in vitro fertilisation. Social science & medicine 1999;48(4):535–46. 10075178 10.1016/s0277-9536(98)00374-8

[20] Zweifel P, Breyer F, Kifmann M. Economic valuation of life and health. Health Economics: Springer; 2009 p. 17–74.

[21] Mitchell RC, Carson RT. Using surveys to value public goods: the contingent valuation method 2nd edn, 2013 Resources for the Future Press, US.

[22] Fox-Rushby J, Cairns J. Economic evaluation. 1st edn, 2005 McGraw-Hill Education, UK.

[23] Kjaer T. A review of the discrete choice experiment-with emphasis on its application in health care 1st edn, 2005 University Of Southern Denmark, Denmark.

[24] Oerlemans LA, Chan K-Y, Volschenk J. Willingness to pay for green electricity: A review of the contingent valuation literature and its sources of error. Renewable and Sustainable Energy Reviews 2016;66:875–85.

[25] Drummond MF, Sculpher MJ, Claxton K, Stoddart GL, Torrance GW. Methods for the economic evaluation of health care programmes. 4th edn, 2015 Oxford university press, UK.

[26] Dominick S. Microeconomics: Theory and Applications. 1st edn, 2003 Ford Ham University, Oxford University Press, New York.

[27] Spiegel U, Gonen LD, Templeman J. Economic implications of in vitro fertilization using willingness to pay. Journal of Public Health 2013;21(6):535–57.

[28] Settumba SN, Shanahan M, Botha W, Ramli MZ, Chambers GM. Reliability and validity of the contingent valuation method for estimating willingness to pay: A case of in vitro fertilisation. Applied health economics and health policy 2019;17(1):103–10. 10.1007/s40258-018-0433-3 30315488

[29] Neumann PJ, Johannesson M. The willingness to pay for in vitro fertilization: a pilot study using contingent valuation. Medical care 1994;32(7):686–99. 10.1097/00005650-199407000-00003 8028404

