# Supplementary material for: Cost-benefit Analysis of IUI and IVF based on willingness to pay approach; case study: Iran
Source: PLoS One. 2020 Jul 14;15(7):e0231584. doi: 10.1371/journal.pone.0231584 (PMC7360055; doi:10.1371/journal.pone.0231584)
Supplement: S1 Fig — (DOCX) [file pone.0231584.s001.docx]

S1 Fig: questionnaire (Persian)

**بسمه تعالی**

پرسشنامه زیر به منظور انجام طرح تحقیقاتی با عنوان **تحلیل هزینه منفعت درمان های ناباروری با استفاده از رویکرد تمایل به پرداخت** می باشد و به صورت مصاحبه انجام و توسط پرسشگر تکمیل می گردد.

**بخش 1 : اطلاعات زمینه ای**

تعریف ناباروری ، دلایل ناباروری در زنان و مردان

ناباروری به عدم توانایی در بچه دار شدن گفته می شود و در اصطلاح به طور معمول به زوجینی نابارور می گویند که پس از یکسال مقاربت بدون جلوگیری زن نتواند باردار شود. امروزه ناباروری در میان زوجین در دنیا شیوع بسیار بالایی پیدا کرده است. این میزان در دنیا حدود 15 درصد است. اما میزان شیوع ناباروری در ایران بالاتر از میانگین جهانی و حدود 22 درصد است. این یعنی از هر 5 زوجی که ازدواج می کنند یک زوج به نوعی با مشکلات ناباروری مواجه می باشد که آمار بالایی است. دلایل مختلفی احتمال ناباروری را در مردان و زنان به وجود می​آورد. بروز هر یک از این احتمال​ها می​تواند بارداری را در زوج دچار اختلال کند. در این شرایط نیز متخصصان پس از 12 بار تکرار تلاش برای بچه​دار شدن، در صورت به دست نیامدن نتیجه به زوج اعلام می​کنند که احتمال ناباروی آنها به یکی از دلایل زیر وجود دارد و آنها برای درمان باید اقدام کنند. گاهی ممکن است اسپرم سالم وجود نداشته باشد یا شکل مناسب را برای باروری نداشته باشد. در برخی شرایط اسپرم فعال و محرک وجود ندارد، یا اسپرم در مسیر رسیدن به رحم از بین برود یا امکان رسیدن اسپرم به تخمک وجود نداشته باشد. گاهی نیز عوامل مناسب برای لقاح وجود نداشته باشد که در این شرایط باید فاکتورهای مناسب در اسپرم و تخمک وجود داشته باشد. در برخی موارد تخمک سالم وجود ندارد یا لوله​های رحمی دچار اختلالی هستند که مانع رسیدن تخمک به اسپرم می​شود. گاهی محیط مناسب برای لقاح وجود ندارد و رحم شرایط لازم را برای باروری ندارد. متناسب با این دلایل و علت​ های مختلفی که برای ابتلا به ناباروری و نازایی در مردان و زنان وجود دارد، شیوه‌های مختلفی برای درمان نازایی و ناباروری وجود دارد که متناسب با علت بیماری توسط متخصص به بیماران توصیه می​شود که عموما این درمانها هزینه های نسبتا بالا دارند و همچنین زمان بر هستند و ممکن است نیاز به تکرار سیکل درمان باشد که صبر و شکیبایی زوجین را می طلبد.

**فرم اخذ رضایت از شرکت‌کنندگان در مطالعه**

این فرم رضایت، گویای حقوق فردی اینجانب به عنوان شرکت کننده در مطالعه " **تحلیل هزینه منفعت درمان های ناباروری با استفاده از رویکرد تمایل به پرداخت** " است که توسط آقای علی درویشی دانشجوی دکتری اقتصاد سلامت دانشکده بهداشت دانشگاه علوم پزشکی تهران انجام می‌شود.

تکمیل پرسشنامه به منظور اطلاع از ترجیحات شما در خصوص موضوع مطالعه می باشد و سوالات توسط مصاحبه کننده پرسیده شده و در حدود 50 دقیقه زمان می برد.

**اینجانب با آگاهی کامل از شرایط می‌دانم که :**

1. شرکت در مطالعه کاملا داوطلبانه است.
2. می توانم از پاسخ دادن به برخی از سوالات امتناع کنم.
3. مجاز هستم پاسخ به سوالات را در هر زمانی که صلاح بدانم، خاتمه دهم.
4. مشخصات من (نام، وضعیت اشتغال و ...) در تمامی مراحل مطالعه محرمانه خواهد ماند.
5. مشخصات من در هیچ فایلی (نوار، دست نوشته یا فایل دیجیتالی) باقی نخواهد ماند.
6. هرجا که لازم است توضیحات کافی به من ارائه می شود.

اینجانب شرایط این فرم رضایت را مطالعه نموده و با اطلاع کامل در این مطالعه شرکت می‌نمایم.

**نام و امضاء شرکت کننده نام و امضاء پژوهشگر**

**2-2 - سناریوهای سطح دوم خدمات ( IUI ):**

**سطح دوم خدمات (تلقیح اسپرم به داخل رحم IUI ) :** در این روش مایع انزال از شوهر زوج گرفته می شود و بعد از شستشو و جداسازی ، اسپرم های زنده بوسیله کاتتر (لوله پلاستیکی) همزمان با تخمک گذاری وارد حفره رحم می شود. تحریک تخمدان قبل از تزریق اسپرم بدلیل دستیابی به شانس بالای حاملگی توصیه می شود. روش IUI روش ساده ای بوده و بیمار بدون ناراحتی همانند گرفتن اسپرم از دهانه رحم این عمل را می تواند تحمل نماید. این درمان را در موارد مختلفی از جمله نازایی با علت نامشخص، بعضی اختلالات اسپرم، وجود موکوس نامناسب در دهانه رحم برای عبور اسپرم و بعضی از بیماران با اختلالات تخمک گذاری می توان استفاده کرد. این روش را معمولا تا چند بار برای موفقیت در یک زوج انجام می دهند. در این روش نیاز به استراحت نمی باشد و بیماران می توانند فعالیت روزانه خود را داشته باشند.

**سناریو 1-**تصور کنید که شما مشکل ناباروری دارید که قابل درمان با IUI می باشد و احتمال موفقیت درمان 10 درصد باشد. با این توضیح که درمان شما تا یکسال یا بیشتر هم ممکن است طول بکشد با این شرایط شما چقدر تمایل دارید بپردازید. آیا شما تمایل دارید 1 میلیون تومان بابت این خدمات بپردازید؟

| بله | خیر |
| --- | --- |
| 1 میلیون و 400 هزار تومان چطور؟ بلی خیر | 600 هزار تومان چطور؟ بلی خیر |
| 1 میلیون 800 هزار تومان چطور؟ بلی خیر | 400 هزار تومان چطور؟ بلی خیر |
| 2 میلیون و 500 هزار تومان چطور؟ بلی خیر | 200 هزار تومان چطور؟ بلی خیر |
| چقدر حاضرید بپردازید؟ | چقدر حاضرید بپردازید؟ |

(WTA): اگر دولت هیچ برنامه ای برای تحت پوشش قرار دادن این سطح از خدمات نداشته باشد و در عوض با پرداخت مبلغی یکجا به صورت یارانه به زوجین پرداخت هزینه های احتمالی را به خود افراد بسپارد، شما چه قدر حاضرید از طرف دولت به صورت یکجا دریافت کنید تا ریسک مالی مربوط به درمان مشکل احتمالی خود در سطح دوم را بپذیرید؟

**2-3 - سناریوهای سطح سوم خدمات (IVF) :**

**سطح سوم خدمات ( لقاح خارج از رحمی** IVF ) : یکی از شیوه​ هایی است که برای درمان نازایی مورد استفاده قرار می​گیرد. در این شیوه لقاح خارج رحمی یا باروری در محیط آزمایشگاه انجام می​شود. این روش در شرایطی که لوله​های رحمی آسیب​دیده باشند یا اختلال در عملکرد آنها مشاهده شود از سوی متخصص به بیمار توصیه می‌شود. این شیوه نه تنها در مورد زنان مبتلا به مشکلات و اختلالات تخمدانی بلکه در برخی موارد درباره ناباروری​ها با علت مردانه نیز به زوج‌ها توصیه می​شود. IVF مشابه IUI ساده و بدون نیاز به بستری شدن بیمار انجام می شود و شامل مراحل تحریک تخمک گذاری، جمع آوری تخمک ها، تهیه و آماده سازی اسپرم مرد، لقاح و رشد جنین ها درمحیط آزمایشگاه، انتقال جنین ها به داخل رحم و تجویز هورمون پروژسترون می باشد.

**سناریو 1-**تصور کنید که شما مشکل ناباروری دارید که قابل درمان با خدمات سطح سوم (IVF) می باشد و احتمال موفقیت درمان 10 درصد باشد. با این توضیح که درمان شما تا یکسال یا بیشتر هم ممکن است طول بکشد با این شرایط شرایط شما چقدر تمایل دارید بپردازید. آیا شما تمایل دارید 4 میلیون تومان بابت این خدمات بپردازید؟

| بله | خیر |
| --- | --- |
| 5 میلیون تومان چطور؟ بلی خیر | 3 میلیون و 500 هزار تومان چطور؟ بلی خیر |
| 6 میلیون و 500 هزار تومان چطور؟ بلی خیر | 2 میلیون و 500 هزار تومان چطور؟ بلی خیر |
| 9 میلیون تومان چطور؟ بلی خیر | 1 میلیون و 500 هزار تومان چطور؟ بلی خیر |
| چقدر حاضرید بپردازید؟ | چقدر حاضرید بپردازید؟ |

(WTA): اگر دولت هیچ برنامه ای برای تحت پوشش قرار دادن این سطح از خدمات نداشته باشد و در عوض با پرداخت مبلغی یکجا به صورت یارانه به زوجین پرداخت هزینه های احتمالی را به خود افراد بسپارد، شما چه قدر حاضرید از طرف دولت به صورت یکجا دریافت کنید تا ریسک مالی مربوط به درمان مشکل احتمالی خود در سطح سوم را بپذیرید؟

**بخش 4 : اطلاعات اجتماعی اقتصادی شرکت کنندگان**

| 1- سال تولد | فرد همسر | |
| --- | --- | --- |
| 2- جنسیت | مرد € زن € | |
| 3- سطح تحصیلات (آخرین مدرک تحصیلی دریافتی) | کم سواد € سیکل € دیپلم €  فوق دیپلم € لیسانس € فوق لیسانس €  دکتری € دکتری تخصصی € سایر € | |
| 5- وضعیت پوشش بیمه | خدمات درمانی ( غیر از بیمه روستایی) € تامین اجتماعی €  خدمات درمانی ( بیمه روستایی) € نیروهای مسلح €  سایر( نظیر صدا و سیما ، شرکت نفت ، بانک ها ، شهرداری ، بیمه روحانیت و غیره ) €  فاقد پوشش بیمه € | |
| 6- آیا تحت پوشش بیمه تکمیلی هستید؟ | بلی € خیر € | |
| 9- وضعیت اشتغال | شاغل € بازنشسته و دارای درآمد بدون کار € محصل یا دانشجو €  خانه دار € بیکار € سایر € | |
| 11- سابقه استفاده از درمانهای ناباروری | قبلاً داشته ام € در حال حاضر دارم € هرگز نداشته ام € | |
| 12- از چه سطحی از درمانهای ناباروری استفاده کرده اید؟ | | دارو درمانی € IUI € ART € |

| 17- میزان متوسط مخارج ماهیانه خانوار شما چقدر است؟ |
| --- |
| 18-میزان متوسط درآمد ماهیانه خانوار شما چقدر است؟ |

"با تشکر از شرکت شما در این مطالعه"
