# Supplementary material for: Cost-benefit Analysis of IUI and IVF based on willingness to pay approach; case study: Iran
Source: PLoS One. 2020 Jul 14;15(7):e0231584. doi: 10.1371/journal.pone.0231584 (PMC7360055; doi:10.1371/journal.pone.0231584)
Supplement: S2 Fig — (DOCX) [file pone.0231584.s002.docx]

S2 Fig: Translated questionnaire (English)

IUI: Scenario 1:

Assume that you are infertile and can be treated with IUI treatment cycles, while the success rate of treatment is 10%, so how much are you willing to pay for it? Are you willing to pay 10,000,000 IRR for it?

| Yes | No |
| --- | --- |
| 14,000,000 yes no | 6,000,000 yes no |
| 18,000,000 yes no | 4,000,000 yes no |
| 25,000,000 yes no | 2,000,000 yes no |
| How much do you willing to pay? | How much do you willing to pay? |

IVF: Scenario 1:

Assume that you are infertile and can be treated with IVF treatment cycles, while the success rate of treatment is 10%, so how much are you willing to pay for it? Are you willing to pay 40,000,000 IRR for it?

| Yes | No |
| --- | --- |
| 50,000,000 yes no | 35,000,000 yes no |
| 65,000,000 yes no | 25,000,000 yes no |
| 90,000,000 yes no | 15,000,000 yes no |
| How much do you willing to pay? | How much do you willing to pay? |
